# The Burden of Alcohol Use: Focus on Children and Preadolescents

**DOI:** 10.35946/arcr.v35.2.09

**Published:** 2014

**Authors:** John E. Donovan

**Affiliations:** **John E. Donovan, Ph.D.,***is associate professor of psychiatry and epidemiology, Western Psychiatric Institute and Clinic, University of Pittsburgh Medical Center, Pittsburgh, Pennsylvania.*

**Keywords:** Alcohol consumption, alcohol use, abuse, and dependence, age of alcohol and other drug use onset, prevalence, alcohol burden, alcohol-attributable fractions, alcohol-related problems, alcohol intoxication, alcohol poisoning, childhood, child, preadolescent, youth, elementary school student, mortality, morbidity, survey, national surveillance data, health and disease, emergency care, treatment, underage drinking

## Abstract

The study of alcohol use by children ages 12 and younger has been very limited. This article summarizes information from U.S. national and statewide surveys on the prevalence of alcohol use among children in grades 6 and lower, data on health conditions wholly attributable to alcohol, the prevalence of children’s treatment admissions for alcohol abuse, and their rates of presentation at emergency departments for acute alcohol intoxication. Factors hampering the estimation of alcohol burden in this population include the lack of ongoing national surveys of alcohol use and problems in children, the hand-me-down nature of alcohol assessments in this population, and the lack of studies to establish whether there is a causal relationship between childhood-onset drinking and morbidity and mortality in adolescence and later in life that would permit determination of alcohol-attributable fractions. This article concludes that although the alcohol burden in childhood is low, it may be augmented by both referred alcohol burden through parental drinking and alcohol abuse and by deferred alcohol burden from longer-term consequences of early use.

The burden of alcohol use usually is expressed as a function of the contribution of alcohol use in a population to morbidity and mortality in that population (Rehm et al. 2010). It is difficult to calculate the burden of alcohol use for middle-school and high-school adolescents (see Patrick and Schulembery, p. 193 in this issue) and nearly impossible to do so for children and preadolescents. There are a number of reasons for this, most of which reflect the early stage of development of the research literature on alcohol use in this young population.

## Factors Limiting Estimation of Alcohol Burden

### The Absence of Recent National Surveillance Data

Chief among the factors inhibiting the estimation of alcohol burden in children and preadolescents is the absence of ongoing national surveillance data. The prevalence of child alcohol use can theoretically be estimated from either adolescents’ retrospective recall of their alcohol use in childhood or from survey research with children.

Retrospective reports of the age at first drink, however, are not very reliable for this life stage. Typically, reported age of onset of alcohol use increases as a function of the age of the adolescents questioned (Bailey et al. 1992; Engels et al. 1997; Johnson et al. 1998; Labouvie et al. 1997; Parra et al. 2003). For example, in the most recent national data from the 2009 Youth Risk Behavior Survey (YRBS), 28.1 percent of 9th graders reported that they drank alcohol before age 13, compared with 14.2 percent of 12th graders (Eaton et al. 2010). These are not cohort effects but rather evidence of “forward telescoping,” as shown by the fact that although the percentages at all grades have declined over time, a similar pattern can be seen in each of the previous YRBS surveys (1991–2007). This pattern also is evident in the 1993–2010 national data from the Monitoring the Future (MTF) surveys (see figures 6 to 20 in Johnston et al. 2011): in every year, less than one-half as many 12th graders as 8th graders report alcohol use initiation by grade 6. Thus, estimates based on retrospective recall are problematic as a summary of the prevalence of childhood drinking.

Direct surveys of children constitute a more appropriate approach for capturing normative data on child drinking. However, of the three major ongoing Federally sponsored national surveys in the United States—the annual MTF survey, the biennial YRBS, and the annual National Survey on Drug Use and Health (NSDUH)—only the NSDUH includes children who are age 12, and none includes children younger than 12. According to the 2010 NSDUH results (Tables 2.15B and 2.16B in Substance Abuse and Mental Health Services Administration [SAMHSA] 2011), 7.1 percent of 12-year-olds had ever had a drink of alcohol (i.e., a can of beer, a glass of wine, or a shot of liquor) in their life, 4.4 percent had a drink in the past year, 1.6 percent had a drink in the past month, and 0.4 percent had consumed five or more drinks on the same occasion.

Despite the absence of children in these ongoing Federal surveillance studies, preliminary information on the prevalence of alcohol use in children has nevertheless been compiled through a comprehensive search of internet sources to locate Nationwide and Statewide surveys of children in grades 6 and below (see Donovan 2007). Based on this review of the four Nationwide and seven Statewide datasets located, it is clear that a substantial number of children have had some exposure to alcohol. Data from the cross-national Health Behaviour of School-Aged Children Study (Nic Gabhainn and François 2000) indicate that in a 1998 national sample of 11-year-old U.S. students, 62 percent of boys and 58 percent of girls had ever tasted alcohol, 8 percent of boys and 7 percent of girls had consumed alcohol at least weekly, and 3 percent of both boys and girls had ever been drunk twice or more. According to the 1999 Partnership Attitude Tracking Study (sponsored by the Partnership for a Drug-Free America), which surveyed a national probability sample of nearly 2,400 U.S. elementary-school students, 9.8 percent of 4th graders, 16.1 percent of 5th graders, and 29.4 percent of 6th graders had had more than just a sip of alcohol in their life. In 2000–2001, the National Survey of Parents and Youth (NSPY) collected alcohol use information on 1,560 9- to 12-year-olds and found that 6.2 percent of 9-year-olds, 5.5 percent of 10-year-olds, 9.2 percent of 11-year-olds, and 15.5 percent of 12-year-olds had had more than a few sips of alcohol in their life. Data on alcohol use in the past year (rather than lifetime) are reported annually by Pride Surveys (see www.pridesurveys.com): according to the 2009–2010 summary of school-district surveys performed across the United States, 4.0 percent of 4th graders, 4.8 percent of 5th graders, and 8.3 percent of 6th graders had drunk alcohol in the past year. Both the Nationwide and Statewide datasets examined showed a decline in the prevalence of child drinking over the past 10 years or so. The datasets located for this review, however, generally are either outdated or nonrepresentative, and their limitations must be recognized in any attempt to estimate the burden of alcohol use in this population. The absence of any recent national survey of alcohol use among children argues for the need to institute ongoing Nationwide surveillance of this population.

It is nevertheless evident, however, that the percentage of children who have experience with alcohol decreases as the intensity of alcohol involvement increases (from a sip or taste to more than a few sips ever to use in the past year, past month, or past week), and that it differs as a function of grade, gender, and ethnicity (see Donovan 2007). Alcohol use rates increased with age, doubling between grades 4 and 6, with the largest jump in prevalence between grades 5 and 6. At each grade level, boys were more likely to have used alcohol than girls. African-American children were nearly as likely as white and Hispanic children to have used alcohol. About one-third as many children reported having had more than a sip of alcohol as reported having had only a sip. In general, around one-third of children who had ever used alcohol reported its use in the past year as well, and use in the past month occurred in only about one-third of those children who reported use in the past year.

There are few current Nationwide data sources on the prevalence of children’s experience of problems attributed to alcohol use that could inform estimates of their wholly alcohol-attributable health conditions (i.e., alcohol dependence and acute intoxication). Several community-level studies suggest that rates of alcohol use disorders are close to zero prior to adolescence (Cohen et al. 1993; Giaconia et al. 1994; Sung et al. 2004). The low number of Nationwide admissions for treatment of alcohol abuse at ages 10–12 bears this out (see figures 14 and 15 in SAMHSA 2008). Patients under the age of 15 constitute just 0.5 percent of those admitted for treatment of alcohol abuse alone and 0.7 percent of those admitted primarily for treatment of alcohol abuse who also had abused another drug (Table 3.2a in SAMHSA 2008).

Likewise, in contrast to adolescents, children rarely present at hospital emergency departments for acute intoxication (alcohol poisoning). In 2009, the rate of visits to emergency departments for acute alcohol intoxication was 5.6 per 100,000 for U.S. children ages 0–5 and 1.0 per 100,000 for children ages 6–11 versus 310.8 per 100,000 for adolescents ages 12–17 (Drug Abuse Warning Network 2010). Of all calls to poison-control centers in the United States in 2009 involving children ages 5 or younger, 2.12 percent of cases involved ingestion of alcohol (Bronstein et al. 2010). This probably is an underestimate, as many children ingested products such as cold medicines, cologne, perfume, aftershave, and mouthwash that contain ethanol (see Vogel et al. 1995).

In summary, there are few surveillance studies of alcohol use and alcohol-related problems among children and preadolescents. The extant data indicate that although the rates of alcohol use are low in this population, substantial numbers of children do have experience with alcohol and the rates of wholly alcohol-attributable health conditions are very low in this population. No evidence has been generated regarding the influence of child drinking on other diseases or injuries within childhood.

### Problems of Measurement

A second major limitation for estimating alcohol burden in this population is the widespread use of “hand-me-down” measures for the assessment of children’s alcohol use. Measures originally developed for use with adults have been modified for use with college students; then modified for use with adolescents; and, finally, modified for assessment of children. Reliance on such hand-me-down assessments has resulted, for instance, in only limited research into sipping and tasting of alcohol despite the fact that this is the most common form of children’s experience with alcohol (see Casswell 1996; Casswell et al. 1991; Donovan and Molina 2008; Johnson et al. 1997).

The hand-me-down nature of child and adolescent assessments is nowhere more evident than in the case of heavy episodic (binge) drinking, a major contributor to adult morbidity and mortality. In adults, binge drinking has been operationally defined as five or more drinks per occasion for men and as four of more drinks per occasion for women (Wechsler et al. 1995); these levels of intake result in blood alcohol concentrations (BAC) of 0.08 percent (the legal definition of intoxication) if consumed within a 2-hour window. Using these definitions for children and adolescents is inappropriate, however, because they weigh less and thus have smaller volumes of total body water than adults. A recent analysis (Donovan 2009) modified the Widmark equation for estimating BAC so it would be more developmentally appropriate. This was done by incorporating formulas for estimating total body water that were derived from children and adolescents and by using ethanol elimination rates derived from child and adolescent presentations for acute alcohol intoxication at emergency departments. BAC estimates were calculated for intake of from one to five standard drinks for boys and girls separately at each age from 9 to 17 to determine how many drinks were required to result in an estimated mean BAC of 0.08 percent or higher. Data from more than 4,700 children and adolescents from the 1999–2002 National Health and Nutrition Examination Survey were analyzed. Girls and boys ages 9–13 had mean estimated BACs of 0.08 percent or higher at three or more drinks, as did girls ages 14–17. Boys ages 14 and 15 had mean estimated BACs of 0.08 percent or higher at four or more drinks, and boys ages 16 and 17 reached this level at five or more drinks. Table 1 summarizes the resulting recommendations for defining binge drinking for children and adolescents by age and gender. Only boys ages 16 or 17 met the adult definition.

In addition to the concern over hand-me-down assessments, there is a lack of consensus on the definition of the various levels of alcohol involvement for both children and adolescents. As is evident in the summary of survey studies above, drinker status was defined variously as consumption of more than a sip, more than a few sips, or a whole drink. This severely hinders the performance of meta-analyses across studies and the description of trends over time. Bacon (1976) noted a similar lack of consensus 35 years ago.

In general, evidence from both test–retest examinations and collateral reports suggests that children’s self-reports of their alcohol use are as valid as adolescent self-reports (Dielman et al. 1995; Donovan et al. 2004). Given their typically low levels of intake and the opportunistic nature of their drinking, misreporting in child reports of their alcohol involvement is unlikely to reflect cognitive overload. More likely, difficulties stem from a lack of familiarity with alcohol beverage types (beer versus liquor, for example) and with estimation of drink volumes consumed. At least one recently developed inventory uses pictorial images to assess alcohol and drug use and their risk factors (see Andrews et al. 2003; Ridenour et al. 2009, 2011).

In addition to making child alcohol assessments more developmentally appropriate and user friendly, surveillance studies of child alcohol use need to be expanded to include questions on the intensity and patterning of their current alcohol use (e.g., frequency of use, usual and greatest intake, frequency of binge drinking, and contexts of use).

### Barriers to Collecting Child Data

Although monitoring the Nationwide prevalence of children’s alcohol use would constitute a step in the right direction, increased research also is needed. It is possible that so few studies have been conducted in this area because of the perception of several barriers to such research (see Donovan 2007). One perceived barrier is that few children drink, so there is little variation to explain. A second is the difficulty of gaining school-district approval to access elementary school populations, necessitating the use of targeted-age directory sampling or household enumeration sampling methods. A third barrier sometimes raised is the misapprehension that parents will be reluctant to consent to their children’s participation in alcohol research.

## Referred Childhood Alcohol Burden Through Parent Drinking

Parents contribute to the alcohol burden of their children in a variety of ways. First, they model drinking behavior for good or for ill. National surveys show that the majority (87.9 percent) of adults in the United States ages 26 and older have ever drank, 69.0 percent drank in the past year, and 54.9 percent drank in the past month (Table 2.37B in SAMHSA 2011). Children learn about alcohol and its effects and usages from observing their parents drinking or from hearing their parents talk about their drinking, as well as from their exposure to drinking in the larger social environment (e.g., relatives, peers and their families, neighborhood events, alcohol commercials on TV and radio, magazine ads, Internet Web sites, social media, and drinking in movies and even in animated feature films) (see Zucker et al. 2008, 2009). Children whose parents drink are more likely to initiate early use (Donovan and Molina 2008, 2011; Hawkins et al. 1997).

Second, parents actively teach their children about alcohol. Children are first introduced to alcohol use by parents or other relatives in a family context (see Jackson 1997; Jahoda and Cramond 1972; Johnson et al. 1997). Such precocious socialization into alcohol use can reflect either Old World cultural beliefs regarding the role of alcohol as food or as a necessary adjunct for celebrations or the belief that introducing children to alcohol use as part of family dinners or events serves to inoculate them from later involvement in problem drinking. Research has not yet established, however, whether learning to drink in a family context actually serves to protect children from developing later alcohol problems. The relevant longitudinal research (Dielman et al. 1989; McMorris et al. 2011; van der Vorst et al. 2010) suggests that this is not the case: prior supervised drinking increases the likelihood of unsupervised drinking and more negative consequences later on. In addition, children who were permitted to drink alcohol at home have been found to show increased alcohol involvement and drunkenness over time (Jackson et al. 1999; Komro et al. 2007). Research also shows that European adolescents, who are more often introduced to alcohol in family contexts, typically are more likely to be involved in binge drinking and intoxication than U.S. adolescents of the same age (Currie et al. 2008; Friese and Grube 2010; Grube 2009).

Third, the home environment is the most popular source of alcohol for children. Among 6th-grade students who had ever had alcohol, the largest percentage (32.7 percent) obtained the alcohol from a parent or guardian the last time they drank (Hearst et al. 2007). Other adults become a more important source of alcohol than parents as children move into adolescence. Greater access to alcohol in the home and greater parental provision of alcohol are associated with greater alcohol intake and problems later on (Komro et al. 2007; van den Eijnden et al. 2011).

In addition to their direct impact on child drinking, parental drinking and alcohol abuse may increase child morbidity and mortality through other means as well. Children also may be placed at increased risk through prenatal exposure to maternal drinking (Jacobson and Jacobson 2002; Mattson et al. 2001; Rasmussen 2005; Richardson et al. 2002; Streissguth et al. 1999); through genetic inheritance of liabilities to alcohol abuse and related addictive behaviors (Schuckit 1994; Sher 1991; Zucker et al. 2003); through alcohol-impaired parenting, abuse, and neglect (Bijur et al. 1992; Dube et al. 2001; Kelleher et al. 1994); and through their adoption of parent-socialized alcohol-specific intentions, attitudes, and expectancies (e.g., Donovan et al. 2009; Handley and Chassin 2009; Tildesley and Andrews 2008), leading to both short-term and longer-term consequences. In addition, children are at risk of injury or death through riding in cars driven by an alcohol-impaired parent: in 2009 alone, 14 percent of the children ages 14 and younger killed in traffic crashes were killed in alcohol-impaired driving crashes, and one-half of these children were passengers in vehicles driven by a driver with a BAC of 0.08 percent or higher (U.S. Department of Transportation 2011).

## Deferred Childhood Alcohol Burden Through Long-Term Consequences

The measurable burdens of child and preadolescent drinking are for the most part postponed into adolescence and young adulthood. Early onset of alcohol use predicts involvement in alcohol problems, alcohol abuse, and alcohol dependence in adolescence (Gruber et al. 1996; Hawkins et al. 1997; Horton, 2007; McGue and Iacono, 2005; Pederson and Skrondal, 1998; Warner et al. 2007). Early-onset drinking also relates to a variety of other problematic outcomes in adolescence, including absences from school, delinquent behavior, drinking and driving, smoking, marijuana and other illicit drug use, sexual intercourse, and pregnancy (Ellickson et al. 2001; Gruber et al. 1996; McCluskey et al. 2002; Stueve and O’Donnell, 2005).

There also is evidence that early initiation of alcohol use affects a number of outcomes in young adulthood as well. These young-adult outcomes include not only alcohol use disorder (e.g., Hingson et al. 2006; King and Chassin, 2007) but also prescription drug misuse (Hermos et al. 2008), substance use disorders (Hingson et al. 2008; King and Chassin, 2007), employment problems (Ellickson et al. 2003), unintentional injuries (Hingson and Zha 2009; Hingson et al. 2000), and risky driving and drinking and driving (Hingson et al. 2002; Zakrajsek and Shope 2006). Retrospective data from adults also have shown a relationship between earlier onset of drinking and lifetime experience of an alcohol use disorders (e.g., DeWit et al. 2000; Grant and Dawson 1997). Research currently is lacking, however, on whether early-onset drinking relates to psychosocial functioning in other young-adult life areas, such as educational, occupational, marital, social, political, and community functioning, and relationship with parents.

As yet, there are few studies of the mechanisms linking early-onset drinking to young-adult alcohol problems and other negative outcomes. McGue and Iacono (2008) see this linkage as emanating from the interrelations between early drinking and other problem behaviors in adolescence (Donovan and Jessor 1985) and the stability of this syndrome into young adulthood (Jessor et al. 1991), which is seen as reflecting both inherited vulnerability and the influence of early problem behavior on the selection of risky social environments. Identification of such underlying mediating mechanisms is an important component of establishing any causal linkage between early-onset drinking and these later outcomes that would inform estimation of their alcohol-attributable fractions (Rehm et al. 2010). The greater the role of mediating variables in this pathway, the smaller the alcohol-attributable fraction is likely to be.

## Conclusions

In summary, there are few surveillance studies of alcohol use and alcohol-related problems among children and preadolescents, a situation that makes estimation of alcohol burden in this population problematic. The available data indicate that whereas the rates of alcohol use are relatively low in this population, substantial numbers of children do in fact have experience with alcohol. With respect to wholly alcohol-attributable health conditions, the available data suggest very low levels of alcohol abuse and acute intoxication among children. The scattered and inaccessible nature of much of this available data highlights the need for better ongoing surveillance of this population. Although these direct assessments imply that alcohol burden in children is relatively low, their alcohol burden is increased through the alcohol use and abuse of their parents, and through the increased likelihood among early drinkers of alcohol problems and other negative outcomes in adolescence and young adulthood.

## Figures and Tables

**Table 1:** Recommended Cut Points (Number of Drinks) for Developmentally Appropriate Definition of Binge Drinking in Children and Adolescents (Donovan 2009)

**Age**	**Boys**	**Girls**
9–13	3+	3+
14–15	4+	3+
16–17	5+	3+
